# INSPIRED but Tired: How Medical Faculty’s Job Demands and Resources Lead to Engagement, Work-Life Conflict, and Burnout

**DOI:** 10.3389/fpsyg.2021.609639

**Published:** 2021-02-10

**Authors:** Rebecca S. Lee, Leanne S. Son Hing, Vishi Gnanakumaran, Shelly K. Weiss, Donna S. Lero, Peter A. Hausdorf, Denis Daneman

**Affiliations:** ^1^Department of Psychology, University of Guelph, Guelph, ON, Canada; ^2^Department of Paediatrics, University of Toronto, Toronto, ON, Canada; ^3^Department of Family Relations and Applied Nutrition, University of Guelph, Guelph, ON, Canada

**Keywords:** work-life conflict, work-family conflict, burnout, job demands, job resources, qualitative, work engagement, physicians

## Abstract

**Background:**

Past research shows that physicians experience high ill-being (i.e., work-life conflict, stress, burnout) but also high well-being (i.e., job satisfaction, engagement).

**Objective:**

To shed light on how medical faculty’s experiences of their job demands and job resources might differentially affect their ill-being and their well-being with special attention to the role that the work-life interface plays in these processes.

**Methods:**

Qualitative thematic analysis was used to analyze interviews from 30 medical faculty (19 women, 11 men, average tenure 13.36 years) at a top research hospital in Canada.

**Findings:**

Medical faculty’s experiences of work-life conflict were severe. Faculty’s job demands had coalescing (i.e., interactive) effects on their stress, work-life conflict, and exhaustion. Although supportive job resources (e.g., coworker support) helped to mitigate the negative effects of job demands, stimulating job resources (e.g., challenging work) contributed to greater work-life conflict, stress, and exhaustion. Thus, for these medical faculty job resources play a dual-role for work-life conflict. Moreover, although faculty experienced high emotional exhaustion, they did not experience the other components of burnout (i.e., reduced self-efficacy, and depersonalization). Some faculty engaged in cognitive reappraisal strategies to mitigate their experiences of work-life conflict and its harmful consequences.

**Conclusion:**

This study suggests that the precise nature and effects of job demands and job resources may be more complex than current research suggests. Hospital leadership should work to lessen unnecessary job demands, increase supportive job resources, recognize all aspects of job performance, and, given faculty’s high levels of work engagement, encourage a climate that fosters work-life balance.

## Introduction

A growing concern for healthcare professionals and the general public is physicians’ ill-being, that is their impaired psychological adjustment, coping, and functioning (Ryff et al., 2006), exemplified by stress, work-life conflict, and burnout. These problems are even more pressing today, as the novel coronavirus (COVID-19) further burdens healthcare professionals and their families (Mahmood et al., 2020). In general, physicians suffer from high stress and burnout (Fuß et al., 2008; Wallace et al., 2009), even more so than community samples (Carta et al., 2017). Physician burnout, in turn, predicts suboptimal patient care and reports of medical errors (Shanafelt et al., 2002, 2010). A key predictor of physicians’ burnout is their high levels of work-life conflict (Langballe et al., 2011).

Despite such struggles with ill-being, physicians also report high well-being, which involves positive evaluations and reactions in daily life (Diener, 2006), such as job satisfaction and work engagement. Physicians have been found to have high work engagement (Mache et al., 2016), and the majority say they would choose their profession all over again (Dyrbye et al., 2011). How is it that physicians experience such high ill-being (i.e., stress, work-life conflict, burnout) and high well-being (e.g., work engagement)? We propose that physicians’ high levels of job resources and job demands play a central role in explaining this contradiction. Furthermore, life circumstances external to, and intersecting with, work roles may create ill-being.

### Work-Life Conflict

Work-life conflict, a form of role conflict, is the struggle that occurs between incompatible demands or pressures from people’s work and non-work life (Greenhaus and Beutell, 1985). According to role theory (Kahn et al., 1964), because each role a person holds requires energy, time, and commitment, the existence of conflicting roles creates pressure for time. Physicians report high degrees of work-life conflict, particularly with their work interfering with their family life (Dyrbye et al., 2011). Further, it has been found that physicians experience greater work-life conflict than other workers (Fuß et al., 2008; Knecht et al., 2010), which occurs primarily due to insufficient time or due to excessive strain at work spreading to home (Knecht et al., 2010).

The high levels of work-life conflict experienced by physicians may exacerbate ill-being, given its many negative personal and job-related consequences. The more physicians experience work-life conflict, the lower their quality of life (Dyrbye et al., 2012), and the higher their stress (Røvik et al., 2007), turnover intentions (Fuß et al., 2008), potential for alcohol abuse, depressive symptoms, (Dyrbye et al., 2012), and burnout (Langballe et al., 2011). Longitudinal research reveals that workers who experience more work-family conflict experience worse negative outcomes, such as lower job satisfaction and negative affect, later (Grandey et al., 2005).

According to the multidimensional theory of burnout (Maslach and Jackson, 1981; Maslach et al., 2001), burnout occurs in response to prolonged stress, and is characterized by exhaustion (feeling stretched thin, and depleted of resources), cynicism or depersonalization (negative and detached feelings toward one’s work) and reduced self-efficacy (feelings of lower self-confidence toward one’s work and feeling less productive and accomplished). Among physicians, work-life conflict predicts emotional exhaustion and depersonalization in particular (Dyrbye et al., 2011). Furthermore, physicians experiencing higher levels of burnout are more likely to provide suboptimal care to patients, commit a medical error, and have suicidal thoughts or die by suicide (Shanafelt et al., 2002, 2010). Given the harmful consequences of work-life conflict and burnout for physicians, an understanding of the processes through which they operate is needed.

### The Job Demands-Resources Model

The job demands-resources model (Demerouti et al., 2001) provides a theoretical understanding of workplace factors that can affect well-being (e.g., engagement) and ill-being (e.g., burnout), as well as the work-life interface (Bakker and Demerouti, 2007). Job demands represent the physical, psychological, social, or organizational aspects of a job that require sustained physical or psychological effort). Conversely, job resources reflect aspects of a job that help employees to manage demands, help achieve work goals, and stimulate growth or development (Bakker et al., 2014). Work engagement involves vigor (high energy, fortitude for work), dedication (sense of significance, pride, challenge), and absorption (engrossed happily in work; Maslach et al., 2001). Job resources are theorized to lead to greater motivation, and demonstrably result in greater work engagement (Bakker and Demerouti, 2007), whereas job demands theoretically lead to stress, and demonstrably result in burnout (Bakker et al., 2014; Montgomery et al., 2015).

### Precursors to Work-Life Conflict: Job Demands

The more people experience job demands, such as longer work hours, greater job insecurity, time pressure, work overload, or emotional demands, the more work-life conflict they experience (Voydanoff, 2004; Peeters et al., 2005); this is presumably because job demands require greater investments of time and energy into work, leaving less for other aspects of life (Bakker et al., 2008). Among physicians, the job demands that have been found to predict greater work-life conflict include: workload, emotional job demands (Bakker et al., 2011), and irregular work schedules (Keeton et al., 2007). Interestingly, medical faculty have been found to experience greater work demands (i.e., longer work hours, less time off, time pressure), compared with physicians in private practice (Linn et al., 1985).

Physicians are also embedded in a culture that normalizes high demands, long hours, going above and beyond for patients, and being selfless and self-sacrificing (Maslach and Goldberg, 1998; Dumelow et al., 2000; Wallace and Lemaire, 2009; Dyrbye et al., 2012, 2013). These cultural norms are perpetuated throughout physicians’ training (e.g., in residency; Meeks et al., 2019), their profession (Maslach and Goldberg, 1998), and/or work setting (i.e., hospitals; Dumelow et al., 2000). In particular, medical faculty struggle with juggling work roles as researchers, administrators, clinicians, and teachers (Wynn et al., 2018). In line with role theory (Kahn et al., 1964), such intra-role conflict or work-work conflict (i.e., when multiple work roles compete for time and energy; Herman and Gyllstrom, 1977) is a well-demonstrated predictor of work-life conflict (Michel et al., 2009). Indeed, it appears that medical faculty who hold more roles have greater work-life interference than those who hold fewer roles (Beckett et al., 2015); and that medical faculty experience worse work-life conflict than other physicians (Linn et al., 1985). Thus, much is known about the job demands that physicians face, which can lead to work-life conflict; what is unknown is how job demands might operate in conjunction with each other, and how the multiple job roles they are associated with may affect work-life conflict.

Greater job demands can lead people to experience greater work-life conflict (Voydanoff, 2004), which can subsequently lead to greater burnout. Further, the negative effects of job demands on physician burnout is mediated by levels of work-life conflict (Montgomery et al., 2006). Indeed, it has been demonstrated that the experience of job demands, work-life conflict, and exhaustion are reciprocal over time (Demerouti et al., 2004). However, the link between job demands, work-life conflict, and burnout may not be straightforward. This is because physicians report large variability in their experiences of the three components of burnout: exhaustion, depersonalization, and reduced self-efficacy (Shanafelt et al., 2015). Thus, the links between job demands, work-life conflict and the different indicators of burnout are not entirely clear.

### Precursors to Work-Life Conflict: Job Resources

Having greater job resources (e.g., work support, schedule flexibility) has been found to reduce work-life conflict, perhaps because they help employees complete their work responsibilities, thus lessoning spillover of work to their home domain (Byron, 2005; Baeriswyl et al., 2016). For instance, the more job resources employees have, such as job autonomy (Schieman et al., 2009; Brauchli et al., 2014), social support (Schieman et al., 2009), and managerial support (Adkins and Premeaux, 2012), the less work-life conflict they report. Meta-analytic evidence supports this notion, as job resources such as work support and schedule flexibility are negatively related to interferences from work to home (Byron, 2005; Michel et al., 2011). Among physicians, having greater job resources, such as colleague-social support, quality leadership, developmental opportunities, feedback, autonomy, and decision-making ability, all predict lower work-life conflict (Mache et al., 2015). In addition, there is evidence that having high job resources can buffer the negative effects of job demands on work-life conflict. For example, among employees with higher job demands, having greater job resources, such as supervisor support, predicts lower levels of work-life conflict (Gu and Wang, 2019), and among medical residents, having higher participation in decision-making mitigates the negative effect of job demands on work-life conflict (Bakker et al., 2011). Thus, there is much evidence that having higher job resources leads to lower work-life conflict.

However, there is also reason to believe that job resources could lead to *greater* work-life conflict through the process of engagement. Having greater job resources intrinsically motivates (Nordhall et al., 2020) and energizes workers, and leads them to experience greater work engagement (Bakker and Demerouti, 2007; Halbesleben, 2010). While engagement is typically associated with positive outcomes, such as better performance and health (Bakker et al., 2014), we propose that engagement could lead to negative outcomes, such as work-life conflict and burnout, because employees could become too engaged in their work, over-involved, and subsequently experience time conflicts between work and home (Byrne and Canato, 2017). Such reasoning is consistent with conservation of resources theory (COR theory; Hobfoll, 1989, 2010), which states that when people have more job resources, they may reinvest their time, effort, and energies back into their work to gain more resources. There is evidence that those who are more engaged in their work perform more extra-role behaviors, which leads to more work-life conflict (Halbesleben et al., 2009). Further research is needed to investigate when and how job resources may lead to a lessening of work-life conflict and when they may lead to more work-life conflict.

### Work-Life Facilitation

A sole focus on work-life conflict hides the potential for a positive experience of the work-life interface and its contributions to well-being. Work-life facilitation occurs when engagement in one domain provides gains (e.g., new skills) that contribute to improved functioning in another domain (Carlson et al., 2006). Greater work-life facilitation has been found to be associated with greater well-being (e.g., job satisfaction; McNall et al., 2010). Among physicians, greater work-life facilitation is related to less exhaustion and disengagement (Langballe et al., 2011). Job resources have been theorized and found to lead to greater work-life facilitation through greater work engagement (Siu et al., 2010). This is because job resources help employees feel engrossed in their work and so they may come home more cheerful or productive after an engaging workday (Bakker and Geurts, 2004). To our knowledge, only one study has investigated this phenomenon among physicians (Langballe et al., 2011), thus, more research is needed to determine the nature and antecedents of work-life facilitation among them.

## Aims and Research Questions

The current study examines medical faculty’s experiences of well-being (i.e., job satisfaction, work engagement, work-life facilitation, empowerment) and ill-being (stress, work-life conflict, burnout). Although all physicians experience threats to their well-being due to high workload, cultural expectations, work-life conflict, and more, we studied medical faculty because they may have particular challenges to explore, given that they hold multiple roles (i.e., researchers, administrators, clinicians, teachers), necessitating that they be “quadruple threats” in their work (Wynn et al., 2018, p. 4), and that they experience greater work-life conflict than physicians in private practice (Dyrbye et al., 2013). A central focus is placed on the processes leading to and resulting from medical faculty’s lived experiences of, and meaning-making about, their work-life interface, given its role in predicting stress and burnout (Røvik et al., 2007; Langballe et al., 2011). We employed thematic analysis because it allows us to analyze faculty’s lived experiences in an in-depth manner.

Our study examined four novel research questions:

(1) Given their work context, how do medical faculty perceive, experience, and make sense of their (a) work-life conflict, and (b) work-life facilitation?
(2) What are the consequences of work-life conflict for medical faculty and their family members?
(3) How do medical faculty’s job demands affect their work-life conflict?
(4) How do medical faculty’s job resources positively affect their (a) work-life facilitation, and (b) do they positively and/or negatively affect their work-life conflict?

## Materials and Methods

### Participants

The sample consists of medical faculty from the largest department of one of the top research hospitals in Canada, which is consistently ranked in the top three of its kind worldwide. Thirty medical faculty participated in this study (19 women and 11 men). Faculty’s average tenure in the hospital was 13.36 years, and all held faculty positions at a prestigious university. Sixty percent reported being married or having a spouse. Participants had between zero and six children and eight faculty (26.7%) had a child younger than 6 years old. We purposefully sampled both leaders and non-leaders, men and women, and those with different job profiles to obtain as diverse a range of experiences as possible. See Table 1 for participants’ demographics.

**TABLE 1:** Participants’ demographic information (*N* = 30).

	Number of Participants
**Gender**	
Women	19
Men	11
**Children**	
< Age 6	8
Ages 6 – 17	7
≥ Age 18	10
Zero children	2
Unknown	3
**Organizational Tenure**	
≤ 4 years	9
5 to 10 years	6
11 to 15 years	5
16 to 20 years	1
≥ 21 years	7
Unknown	2
**Leadership**	
Leadership role (e.g., division head)	11
No leadership role	19
**Professor Rank**	
Assistant professor	13
Associate professor	9
Full professor	7
Lecturer	1
**Predominant Job Role(s)**	
Researcher	4
Researcher, Clinician	4
Educator, Clinician	5
Clinician	17
**Division**	
Neurology	5
Pediatric Medicine	5
Emergency Medicine	4
Adolescent Medicine	3
Cardiology	3
Nephrology	3
Hematology/Oncology	2
Endocrinology	1
Immunology	1
Neonatology	1
Rheumatology,	1
Unknown	1

### Interview Guide

The interview guide was developed by drawing on previous research and theory, such as the job demands-resources model (Demerouti et al., 2001), role theory (Katz and Kahn, 1978), work-life conflict (Greenhaus and Beutell, 1985; Voydanoff, 2005), and work-life facilitation (McNall et al., 2010). In addition, our research questions and interview guide were created in collaboration with S.W. and D.D., who are medical faculty at the hospital. The interview guide was piloted with two additional medical faculty from the hospital and was refined based on their feedback.

The interview guide began with general questions (e.g., “Can you describe the work that you do?”), then narrowed to more specific topics such as job demands, job resources, stress, and the work-life interface (e.g., “Have your work and nonwork lives had any positive effect on each other?”). It also included follow-up or probing questions. Each interview followed the interview guide; however, when new areas of interest emerged from participants’ responses, new interview questions or probes were devised (e.g., “What do you find more stressful, demands at work or demands at home? Why?), and the interview guide was amended. See Appendix for the final interview guide.

### Procedure

The study received formal ethical approval from both the hospital’s and R.L.’s institutional research ethics boards. Faculty were invited through email to voluntarily participate in a study examining their stress and work-life conflict to inform the hospital about faculty wellness. Individual interviews were conducted by V.G. in the privacy of faculty’s offices at the hospital. Participants were introduced to the study, assured confidentiality of their responses, and asked to sign the consent form. The audio-taped interviews ranged from 40 to 60 min and were transcribed verbatim.

Twenty interviews were conducted with preliminary analyses (open coding) occurring in tandem with data collection. Following this initial data collection, researchers paused to discuss findings, and it was determined that, because we were still establishing new codes, saturation had not been met. Another recruitment email was sent to the same department of medical faculty. Ten more interviews were conducted, until saturation was met.

Following completion of the interviews and preliminary analyses, six feedback sessions were conducted with 13 participants to garner their responses to our initial findings. Overall, faculty reported that the findings were consistent with their experiences but wanted to reiterate or expand on some key points, such as the hospital culture, and the perceived valuing of research over clinical work. Their feedback may have made these issues more salient during the data analysis and theme creation process.

### Data Analysis

The coding manual was initially developed *a priori*, using previous literature, theory, and research questions as guides (e.g., the job demands-resources model; Demerouti et al., 2001; role theory; Katz and Kahn, 1978; work-life conflict; Voydanoff, 2005, work-life facilitation; McNall et al., 2010). However, the coding process was iterative, as the manual was updated to reflect new codes that emerged from the interviews (Boyatzis, 1998). Thus, the coding was both deductive (theory-driven) and inductive (data-driven). An example of a deductive code is workload (given existing measures of job demands; Bakker et al., 2011), and an example of an inductive code is opportunity for development (given existing measures of job resources). In addition, some excerpts of data could also be coded multiple times. The quote, “the things that you regret are the things that you didn’t do…the school concerts you missed and the you know the opportunities to see the growth and development of your family,” was coded as guilt/resentment, and work conflicting with life. The interviews were also coded for both semantic (i.e., explicit) and latent (i.e., implicit) content. The coding manual contained the code names, definitions of each code, and sample quotes to illustrate the codes; see Table 2.

**TABLE 2:** Themes, subthemes, and examples of codes.

Themes	Subthemes	Examples of codes	Description of example code
**Research Question 1:** Given their work context, how do medical faculty perceive, experience, and make sense of their (a) work-life conflict, and (b) work-life facilitation?			
**(a) Work-Life Conflict**			
Experience of high levels of work interfering with non-work life	Lack of work-life balance	Work conflicting with life	Participation in the work domain (e.g., when work is brought home) interferes with the non-work domain
		Life conflicting with work	Participation in the non-work domain (e.g., elder care and childcare responsibilities) interferes with the work domain
	Prioritization of work due to permeability of home boundaries	Permeable (life) boundaries	Work easily encroaches into the non-work domain due to a lack of restrictions
Experience of low levels of work-life conflict	Managing work-life conflict	Low levels of work-life conflict	Experiences of work-life conflict that are of low intensity or that happen only occasionally
	Coping through cognitive reappraisals	Cognitive reappraisals	Acceptance that interference between work and non-work domains will sometimes occur; normalization of experiences of conflict between work and non-work domains
**(b) Work-Life Facilitation**			
Vague experience of work-life facilitation	Wishful platitudes	Difficulty in describing work-life facilitation	Descriptions of work-life facilitation that were cursory, vague; consisted of platitudes
Specific experience of work-life facilitation	Positive effectsbetween work and life	Facilitation from work to life	Participation in the work domain results in positive developmental, affective, or capital gains for functioning in non-work domain.
		Facilitation from life to work	Participation in the non-work domain results in positive developmental gains for functioning in work domain.
**Research Question 2:** What are the consequences of work-life conflict for medical faculty and their family members?			
Consequences for both work and home domains	Stress	Distress	Experience of psychological distress resulting from work-life conflict
	Exhaustion and fear of burnout	Fear of burnout	Faculty express fear of experiencing burnout (may be experiencing symptoms already)
Consequences for home domain	Lack of self-care	Sleep deprivation	Lack of sleep; repercussions for physical/mental wellness;
	Impaired family relationships	Impact on spouse	Negative effect of work-life conflict on one’s spouse or partner
		Impact on children	Effects of work-life conflict and/or work on one’s children
	Negative emotional consequences	Guilt or resentment	Feeling guilty as a result of work-life conflict; resentment of workload or missing out on home domain activities
Consequences for work domain	“Mommy track”	Cut Down to part-time	Reduction of work hours (from full-time to part-time) to manage work-life conflict and/or work responsibilities
**Research Question 3:** How do medical faculty’s job demands affect their work-life conflict?			
Coalescence of demands to create work-work conflict that leads to work-life conflict	Hindrance demands	Workload	Overwhelming amount of work that includes long work hours, lack of time to do work, time pressure to complete tasks, fast or unrelenting pace of work, inability to take a break, and menial, administrative paperwork
		Poor information technology (IT)	Difficulties with the IT in the hospital (e.g., slow computers, outdated technology)
	Cultural expectation of constant availability	100% availability expected	Expectation to always be available for work (e.g., responding to messages and emails at any time; travel for work responsibilities)
	Work-work conflict	Intra-role conflict	Faculty try to juggle multiple work roles (i.e., research, clinical, education/teaching, and/or administrative), which results in competition for time/energy
		Intra-role conflict feeds into inter-role conflict	Inability to meet all work roles in a full day of work means that work is brought home. Specifically, work that must be conducted at the hospital (e.g., clinical duties and teaching) forces research to be conducted during non-work time
	Culture of excellence		Expectations for faculty to always perform to levels of excellence
	Perceived prioritization of research	Clinical work undervalued	Belief that clinical work is not rewarded by the hospital; being a good clinician not good enough; faculty put in extra work and time to also perform well in their research and teaching roles in order for their work to be valued
		Research pressure	Pressure to conduct high-quality research, publish in top-tier outlets, and obtain research funding
Legitimization of work-life conflict	Rationalization	Sense of inevitability	Belief that work pressure is an inherent part of the job/of working at a top research hospital, what they signed up for
**Research Question 4:** (a) How do medical faculty’s job resources positively affect their work-life facilitation, and (b) do they positively and/or negatively affect their			
work-life conflict?			
**(a) Job resources and work-life facilitation**			
Job resources and work-life facilitation	Job resources facilitate spending more time at work	Salary allows for childcare	Faculty have good salaries that allow them to afford childcare, which can enable them to spend more time at work (knowing that their children are being cared for)
**(b) Job resources and work-life conflict**			
Job resources help to lessen work-life conflict	Supportive job resources	Support from superiors and coworkers	Support from others to manage work-life conflict, such as coworkers’ covering shifts, advice from mentors/supervisors
		Organizational support	Faculty receive support that helps them do their job more effectively; includes: working with high-quality allied health staff (e.g., nurses), competent administrative support, adequate funding, adequate physical resources, status of the hospital
Job resources indirectly lead to more work-life conflict	High levels of stimulating job resources	Opportunities for development	Faculty have opportunities for personal and/or professional development; includes: challenging and interesting work, dynamic work with a variety of tasks, opportunities to innovative, learn, develop a niche (e.g., a narrow specialty)
	Sense of meaning and gratitude	Opportunities for impact	Faculty have opportunities for to make a difference in others’ lives, such as, treating/caring for patients and their families; teaching and training trainees, students, and residents
	Feelings of empowerment and motivation	Excellent colleagues	Faculty have opportunity to work with others (colleagues, collaborators), who are expert, highly invested in their work, and committed to excellence

The coding process followed thematic analysis guides and recommendations (Braun and Clarke, 2006). Each transcribed interview was coded by R.L. and V.G. in NVivo 10; extensive notes about the coding process and decision-making were kept by both analysts, who engaged in continuous consultation with each other and L.S.H. When disagreements arose, they were resolved through discussion with all analysts (R.L., V.G., L.S.H.).

R.L. and L.S.H. created themes by connecting the codes (Crabtree and Miller, 1999) that address the research questions. Theme creation was conducted through extensive discussion, careful consideration of the interview data, the context in which the interview data was experienced and described, the corresponding codes, and the description of the codes. Importantly, we looked for similarities as well as differences across the interview responses, as well as across different groups, for example, physicians of a specific job-profile, or among women versus men.

#### Reflexivity

Reflexivity in qualitative research is a process by which researchers are aware of and reflect on their role as the researcher and how their experiences, biases, preferences, and relationships can influence the research and the research process (Korstjens and Moser, 2018; Dodgson, 2019). R.L., L.S.H., V.G., D.L., and P.H. were outsiders to the hospital, as well as non-physicians, therefore they have not directly experienced the caring for patients and patients’ families, medical training, and work in medical environments. Therefore, R.L., L.S.H., and V.G. consulted with S.W. and D.D. (i.e., medical faculty at the hospital), throughout the research process, as they offered context and insight to the experiences of physicians. One of the three coauthors who conducted the data analysis, L.S.H., is a faculty with multiple work roles and a parent, creating a risk of contamination; however, the other two (R.L., V.G.) are neither faculty, nor parents, thus, a balanced perspective was brought to the data. It is possible that S.W. and D.D., as organizational members and leaders in the hospital, may have been motivated to interpret the findings through a positive lens. However, these coauthors were not involved in the data analyses nor in the creation of themes.

Further, R.L. and L.S.H.’s data analyses were informed by psychological theories and models (e.g., role theory, the job demands-resources model, COR theory). Drawing from previously established theories and models may have led to confirmation bias in the data analysis. However, we purposefully engaged in discussions about what participants said that was divergent and unexpected from these theoretical perspectives. We also deliberately explored variation among participants in their reported experiences.

## Findings

### Research Question 1

Given their work context, how do medical faculty perceive, experience, and make sense of their (a) work-life conflict, and (b) work-life facilitation?

#### Work-Life Conflict

Theme: Experience of high levels of work interfering with non-work life

Many faculty reported struggling tremendously with integrating their work and home lives and described high work-life conflict. One faculty said, “I think people talk about work-life balance and most of us don’t have……there is no balance, it’s just all work. I kind of am working 100% of the time, even if I’m not working, I’m working…I do feel like I have to spend all day Saturday and all day Sunday working and every evening because of the things I have taken on that have to get done. But that in turn leads to, you know, less social interactions, less interpersonal pleasures, less possibility to do other things.” Faculty were much more likely to experience work interfering with their non-work life than vice versa. When discussing family needs, one faculty shared, “I never let it interfere [with work]. My family was on the block, they always paid the price.” Prioritization of work over home appears to reflect that home boundaries are more permeable than work boundaries, thus, it is easier to have work eat into home time than vice versa (Frone et al., 1992). Indeed, one interviewee revealed that they receive, “a level of tolerance [from family] that allows me to be fairly extreme on the commitment side of the business.” Such prioritization may also reflect the primary salience of their work role—in particular their role as a clinician that requires self-sacrifice for their patients (Maslach and Goldberg, 1998).

Theme: Experience of low levels of work-life conflict

In contrast, a few faculty explicitly reported lower levels of work-life conflict, stating that they only experience their work encroaching on their home life “sometimes,” such as when they are on call or because they have fewer at-home responsibilities. For instance, one interviewee said that managing work and home life is “just a matter of organizing” and moving schedules around, while another reported that, as a result of her children being older, she was able to fully throw herself back into her work.

It appears that some faculty attempt to manage their experiences of work-life conflict by engaging in cognitive reappraisals that facilitate the reinterpretation of situations to change their emotional impact (Buhle et al., 2014). Some of these faculty normalized their levels of work-life conflict, despite negative personal implications, “I frequently have to make a choice – or believe I have to make a choice – which favors work as opposed to family…That obviously has an impact on your wellbeing. It’s amazing how tolerant you become of anything, no matter how extreme it is when you do it all the time. So, it doesn’t feel like it’s an aberration. It just feels like it’s normal. It’s part of what you do. It’s part of who you are.” Others rejected the notion that work and home life can conflict, suggesting that sharp distinctions between work and home should not be drawn so that they “don’t get so upset that work goes home and home comes to work.” One participant shared, “I have been thinking about work-life integration and trying to not be fussed about two separate buckets too much…by conventional standards I have enormous encroachment of work…answering emails, looking at my phone, reading, catching up on work after hours. I think [my spouse is] aggravated by the chronic nature of taking work home.” It appears that these faculty engage in cognitive reappraisals to deal with high levels of work-life interference, whereby they reject the notion of “conflict” in order to be less resentful when work and home overlap because no expectation for separation is violated.

#### Work-Life Facilitation

Theme: Vague experience of work-life facilitation

When asked if their work and non-work lives had any positive effects on each other, faculty mostly provided responses that were vague, short, non-illustrative, and containing wishful platitudes. One faculty responded, “you hope that you get satisfaction in both and so they would feed positively into each other,” while another said, “people that are happy at home and their home lives I think are happy at work, they’re better workers, right? I think it has to be in that direction.” Typically, work-life facilitation was not a well-articulated phenomenon, especially in comparison with the vivid, detailed, and sometimes painful stories about work-life conflict.

Theme: Specific experience of work-life facilitation

However, some physicians clearly experienced work-life facilitation. Some faculty described facilitation from their work domain to their home domain. For example, one respondent shared, “I feel like I’m a role model to my children…by having a career and showing them what hard work and going to school and what all that means and why it’s important,” reflecting on how their dedication to and satisfaction from their job provides a good example for their children. Another faculty discussed how skills they learned at work transferred to their home domain, sharing that, as a doctor, he learned to place great focus on patients so that a 5-min visit may seem like 30 min to the patient and how, “it’s the same thing with your wife or kids.” Further, some discussed how their home roles can facilitate work roles, for example, how being a parent enabled them to communicate better with patients and their families – “I think being a mom has allowed me to be a better pediatrician. I can really relate to parents, their fears. I have a lot more patience for families, more knowledge. I can relate to kids better.”

### Research Question 2

What are the consequences of work-life conflict for medical faculty and their family members?

Theme: Consequences for both work and home domains.

The greatest expressed consequences of work-life conflict were stress and exhaustion, the main component of burnout, which is feeling overextended and depleted of one’s emotional and physical resources (Maslach et al., 2001). One interviewee stated that they were, “always feeling sort of pulled in two different directions; trying to be a mom and trying to be a professional. I’m sure it takes a toll on me more than I’m willing to admit.” Faculty also described their experiences as “exhausting.” One relayed, “you feel like you [are] so drained at work that you didn’t have much to give when you went home.” Interestingly, no faculty described themselves as actually feeling burnt out; that is, they did not also report feelings of depersonalization (negative and detached feelings toward one’s work) or reduced self-efficacy (feelings of lower self-confidence toward one’s work and feeling less productive and accomplished, which are the other facets of burnout (Maslach et al., 2001). Rather, for some, there is a fear that they cannot escape burnout long-term. One respondent worried that their workload and pace would “catch up to me,” while another shared, “It’s not clear how long people can sustain working at 115% or 120%…I watch people begin to burn out and I can’t help them and that’s very frustrating. I’m afraid what will happen is that we’ll lose good people.”

Despite being health care providers, faculty reported ignoring their own health as a result of work-life conflict. One respondent mentioned ignoring regular medical checkups due to work and family responsibilities, and another stated, “If a bunch of things are piled up and I can’t get to them because… I’m on service or… even with like a 12-h day, I come home, and you know, the kids are in bed and I’m right back at the computer to do more work…that’s not healthy. I think the long hours, I have really compromised things like you know, fitness and personal time and hobbies and down time.” Many faculty also described work-life conflict as leading to sleep deprivation. One faculty shared that they sleep about 4 h per night, while another explained how they worked, “a 12-h shift, on pretty little sleep, and that makes me very anxious because…I’m sort of in charge [in an acute care setting], and I worry that my lack of sleep could potentially affect my decision making.” Thus, the dangers of work-life conflict can also be a cause for concern for patient care and for the hospital.

Theme: Consequences for home domain.

In addition, the consequences associated with work-life conflict extended to some faculty’s children and spouses, as faculty reported frequently missing out on family dinners, attending children’s activities, and spending quality time with friends and family in order to work. A faculty member shared: “The children, that was hard, because you have to outsource everything, you’re just outsourcing all the time. And so, one of my children…said one time they were asking for donations to [the hospital], she stepped back, became a little red in the face and she said I gave my mother to [the hospital]. That’s a real tear-jerker and it’s true.” Another said that her daughter “insists she’s in therapy” because she was predominantly cared for by others while growing up. Although relationships with children can be strained as a result of work-life conflict, this is particularly true for partners or spouses. One respondent asked, “What do I do when my wife is yelling at me because I’m late again?” Further, some faculty members said that they don’t know their spouse well anymore because, although they put in considerable effort when it came to their children, their marriage was “easy to compromise” for work.

In contrast, a few faculty reported that their prioritization of work over family may have a positive outcome on their children because they develop skills (e.g., autonomy, independence) that they might not otherwise. One faculty explained that while they recognize that they haven’t spent enough time with their children growing up, “having a working mom really facilitates their independence,” while another faculty shared, “I haven’t spent as much or enough time with my boys growing up. It has certainly made them independent in the extreme.” Thus, it appears that some faculty engage in cognitive reappraisals of their situation, such that they view their potentially negative experiences of work-life conflict in a positive light.

Faculty also experienced negative affect as a consequence ofwork-life conflict. For some, the sacrifice of work overtaking alltheir time was a source of resentment, as work was seen to cause them to be absent from the lives of people they care about, for example, “It is hard to disconnect from work. So there are times when I resent that…if one of the kids has a hockey game or something and I can’t go because I am here with a patient or student after hours…you know, you can resent that for a little while.” Interestingly, only women in our sample shared experiences of work-family guilt, that is, the negative feeling that arises when one’s desired level of participation in a role is less than one’s actual level of participation (Hochwarter et al., 2007). For instance, one woman shared, “the things that you regret are the things that you didn’t do…the school concerts you missed and the, you know, the opportunities to see the growth and development of your family.”

Theme: Consequences for work domain.

As a result of work-life conflict, some faculty reported considering or temporarily changing to part-time employment to manage these aspects of their lives better. Only women mentioned either working part-time or wanting to change their status to part-time. For instance, one faculty described her 12-h workdays and said, “You do that a bit and all the wheels fall off. The children, grandchildren, certainly the husband…You’d think you’d have some sort of detachment but no. I just can’t put in those hours anymore.” In addition, another woman faculty shared, “The reason why I work [less than full-time] was so I could take my kids and pick my kids up from school 1 day a week…I took a 20 percent pay cut for 10 years of my career for that.” Thus, as a result of work-life conflict, women faculty are potentially feeling forced onto the “mommy track,” whereby they sacrifice their career progress and promotions in order to be present for their children.

### Research Question 3

How do medical faculty’s job demands affect their work-life conflict?

Theme: Coalescence of demands to create work-work conflict that leads to work-life conflict.

While some demands had a direct relation to work-life conflict (e.g., shift work created work-life conflict because faculty were either working or sleeping when family members were at home), by and large we found that job demands coalesced in a manner that drove faculty to work long hours resulting in work-life conflict. One faculty described this phenomena: “There’s always more work to be done, so it’s almost like a never-ending pit. You just try to do as much as you can and stay sane.” Faculty experienced a number of job demands that hindered their ability to conduct their work efficaciously and efficiently, or even created more work for them, such as poor equipment, unsupportive staff, and outdated, slow IT systems. These demands added to faculty’s already excessive workload, which was called a “constant, unrelenting, bombardment of tasks and requests.” These types of demands led to greater work-life conflict because they required faculty to stay at work late or bring work home.

Medical and hospital culture expectations added to workload demands. For instance, faculty are expected to always be available for contact, to be available for early morning and evening meetings, to work until late at night, to self-sacrifice, and to give their work their all, as “there is a general philosophy that if you aren’t working 120% that you are not being productive, it’s a real strong sense that you should be working at home at night. You should be working every night till 2:00 in the morning…That’s a cultural thing that’s probably really not positive at all for people.” Indeed, more than one faculty said that they can get so busy that they do not go to the bathroom, and one participant shared a story of working out at the onsite gym (provided for work-life balance) only to be greeted by 10 emails saying, “Where were you?” at the end.

Faculty’s struggle with their excessive job demands should be considered in the context of their multiple work roles (i.e., research, clinical, teaching, administration). Duties associated with each role often compete with each other for time and energy in an overloaded workday. One faculty member described their experience: “Administration eats a large chunk of my day and my week. Research expectations are still relatively important, even though there’s a large administrative component to my work. Expectations around education, both formal and informal. They all eat into the day. They’re all incredibly important. Research and education. That’s part of our portfolio because it’s a given that if you work in an academic unit, you perform, not just clinically but you perform administratively…So keeping all the balls in the air and continually juggling.”

For faculty, there is work associated with their clinical, teaching, and administrative roles that can only be completed at the hospital (e.g., clinical consults, rounds, teaching residents, administrative meetings), and despite “protected time” (i.e., time protected for research), clinical duties often take up the entirety of a typical workday. However, research is highly valued and prioritized, as it leads to grants, funding, and promotions; as such, faculty often have no choice but to take their research work home to complete. One faculty shared, “Everything is fast paced. The clinical work is certainly fast-paced, and I think that can take over the other work…If you don’t sort of try to make time for it, your research won’t get done and teaching sort of falls secondary.” Thus, not only do faculty experience extremely high job demands, these job demands also compete with each other for time and energy, leading to experiences of stress, and work-life conflict for faculty.

Overall, faculty felt they were expected to be exemplary in all of their roles, particularly their research role. The culture emphasizes a standard of “excellence beyond excellence;” one faculty stated that negative outcomes such as stress and work-life conflict are “inevitable [in a place like this] because of the ridiculously high expectations. That’s the tone of the institution,” adding that people are selected because they are “driven and motivated and succeed, and that kind of perpetuates the expectations and demands and stress.” However, faculty were particularly burdened by the pressure to perform academically because, “it is not enough to be a good clinician” and, “it is still research that is most valued.” The hospital’s culture prioritizes research by celebrating research superstars, who are plentiful, and an awareness of “have” versus “have-not” divisions adds to a sense of differential value or status.

The apparent emphasis on research is reinforced through multiple performance reviews, One faculty explained, “Every year…we have to update our CV, reporting changes that we’ve accomplished over the last year…the template that they give you to report only really wants to know about papers that you have published, conferences you’ve been invited to speak at, and research-oriented projects. So, for those of us who are not research-oriented, it really makes you feel like you’ve pretty much done nothing for the last year and that can be quite frustrating.” The performance reviews were widely acknowledged by respondents to reward research over clinical, teaching, and administrative work. However, a few faculty—all of whom had a heavy research profile—rejected the notion that research productivity is valued above all else and framed such a view as a “mythology” and a problem of the past. Regardless of their veracity, to meet these supposed standards, faculty devote considerable time to conducting research, writing grant applications, giving talks at conferences, and publishing papers, which comes at the cost of their home time. One interviewee explained, “the promotion process [is] standardized for a select group of people; generally, people that are very research focused…and it particularly is not aligned with someone who is a parent.” As a result, faculty experience this ‘perfect storm,’ wherein coalescing job demands lead to work-life conflict. This storm occurs when, in a context that expects excellence for all aspects of work, faculty’s multiple teaching, administrative, and clinical duties vie for limited time and attention during hospital hours and push out time that is meant for research, which needs to be prioritized. As a result, research-related work must be taken home, resulting in work-life conflict.

Theme: Legitimization of work-life conflict

Notably, some respondents perceived work demands and work-life conflict differently. Some participants rationalized work demands as an inevitable and expected part of the job, given their specialty or given their position at a top research and teaching hospital. One respondent said, “I don’t have a lot of time for people who whine about their life-work balance…it’s a personal choice you make,” while another said, “It’s silly to complain about [being on] call because I think it’s just part of the job…you’ve made that choice…[I chose a sub-speciality] that probably wouldn’t allow me to have a separate personal life.” Thus, some faculty appear to legitimate work demands and the effect on their own and others’ non-work life.

### Research Question 4

(a) How do medical faculty’s job resources positively affect their work-life facilitation, and (b) do they positively and/or negatively affect their work-life conflict?

#### Job Resources and Work-Life Facilitation

Theme: Job resources lead to greater work-life facilitation.

Given the lack of discussion of work-life facilitation, it was not possible to draw strong connections to job resources. However, a few respondents discussed how good salaries allow them to go on trips with their families or afford expensive hobbies, and several mentioned how their pay allowed them to afford childcare, which aided faculty to spend longer hours at work. One interviewee said that they’d be divorced if they didn’t have the employed household help, adding, “the domestic support is huge.” Thus, pay allows faculty to manage their work-life conflict and it enables them to throw themselves into work even more.

#### Job Resources and Work-Life Conflict

Theme: Job resources help to lessen work-life conflict.

It appears that having more job resources that help faculty get their work done or that help provide social support, which we term *supportive job resources*, lessen work-life conflict. Supportive job resources – such as financial support from the hospital, competent administrative support, adequate physical resources, schedule flexibility, support from mentors/supervisors, as well as co-worker support to help cover shifts, train residents, and help with curriculum development – all lighten faculty workloads and help to streamline their work. One faculty commented on the collegiality within their division, “I think that if you need help with something, people will help you out. If you need help [with] last minute with things, people will help you. If my children were sick today, someone would do my work for me.” This reduces the need to work late or bring work home, thereby lessening faculty’s work-life conflict, even in the face of high job demands.

Theme: Job resources indirectly lead to more work-life conflict

In contrast, it appears that having more, what we term, *stimulating job resources*, lead to *greater* work-life conflict. Stimulating job resources are those job resources that provide opportunities for growth and development, and opportunities for meaningful and impactful work. When faculty were asked what about their job is stimulating or motivating, almost all faculty answered with the vast opportunities to have interesting, dynamic, and varied work that is challenging and allows for innovation and specialization. One interviewee said, “the variety of what we get to do and the opportunity for growth and development and constant challenge…I don’t think you can find a parallel anywhere,” another stated, “It’s very interesting work that I do. It’s different every day. You never know what you’re going to get. I learn something new every day. It’s not static at all.”

Faculty also enthused about how their job gives them opportunities to affect others’ lives. One faculty said, “To be able to [help patients]…to be a part of families’ lives when they’re going through crisis and to be able to help… there’s just nothing like it…Like, who gets to do this?” Further, faculty received immense satisfaction from working with trainees and felt the significance of training the next generation of physicians. Putting it all together, one faculty member shared, “I think it’s an amazing privilege to wake up every morning and come here and get to do what I do and see what I see and interact with the people I interact with, both co-workers and patients and families and trainees, all of the above. To get a paycheque for it at the end of the day is unbelievable.” It appears that the incredible, stimulating job resources that faculty have, such as opportunities for challenge and growth and the opportunities to make a difference in others’ lives, provide faculty with a sense of awe and gratitude. In addition, they also felt privileged to work at such an elite institution with such highly regarded colleagues.

Together, these stimulating job resources led faculty to feel empowered, that is competent, autonomous, and feel their work has meaning and impact (Spreitzer, 1995). Such high levels of empowerment appear to lead faculty to “love” their job, and be highly engaged, despite all of the job demands discussed earlier. That such job characteristics can lead to motivation is consistent with self-determination theory (Ryan and Deci, 2000) and theories of psychological empowerment (Spreitzer, 1995), which are popularized in the book “Drive” (Pink, 2009). To illustrate, one respondent shared, “I love coming to work. I love my job. I feel really lucky. And even though the job is intense and demanding and eats into my private time, I wouldn’t do that if I wasn’t enjoying the work…So, [being passionate] helps me, motivates me, to do more and do better.” Indeed, such high work engagement may lead to work-life conflict, as it leads faculty to spend exorbitant amounts of time at work, bringing work home, and working during home time meant for family or recuperation. As one participant said, “[Work] is really stressful, but I really am passionate about this and happy to do it so, I’m gonna buckle in.” Thus, stimulating job resources seem to lead faculty to be so engaged with their work that they choose to invest more into their jobs, even at the expense of their home life.

## Discussion

The current study examined novel insights leading to and resulting from physicians’ experiences of their work-life interface. Although our findings highlight the different ways faculty make sense and meaning of their experiences; some dominant themes emerged. In contrast to the vivid and dire descriptions of their work-life conflict, faculty had little to say about work-life facilitation. We found that physicians’ job demands interact with each other to contribute to work-life conflict, stress, and exhaustion. Further, the effects of job resources on work-life conflict appear to depend on whether they are supportive or stimulating in nature. Faculty described supportive job resources (e.g., coworker support) as protecting against work-life conflict. In contrast, a pattern was revealed whereby stimulating job resources (e.g., challenging work) lead to greater work engagement, more time investment in work, and subsequently *greater* work-life conflict. Finally, we found many consequences to faculty’s experiences of work-life conflict for themselves, their careers, and their families.

### Theoretical Implications

First, in contrast to the job demands-resources model, the results of the current study reveal that the effects of job demands are neither unique nor additive (Michel et al., 2009). It appears that faculty’s work demands operate in a conjunctive manner—multiple and competing work roles, insurmountable workloads, a need to be ever-present, a culture of excellence, deeply embedded norms to put patients above themselves, and a need to be a productive researcher—coalesce to drive faculty to work long hours, bring work home, miss family events, and neglect self-care. Given that medical faculty’s job demands had a coalescing and cascading effect on each other to then affect work-life conflict (i.e., they interact with each other), we posit that if any one of these demands were to change, the pressure from the other demands would be greatly reduced, and this could diminish work-life conflict significantly. The results of the current research highlight a potential limitation of the job demands-resources model and suggest that it should be expanded to include the potential interactive effect of job demands with other job demands on experiences of stress, work-life conflict, and burnout.

Second, and relatedly, we found that intra-role conflict or work-work conflict (i.e., when faculty’s multiple work roles compete for time and energy) can lead to work-life conflict, and importantly, the process through which this operates depends on the permeability of role boundaries and on the salience of the roles. While many faculty felt strong pressure to prioritize their research role; clinical, administrative, and teaching duties took up the majority of a long workday, as these duties must be completed at the hospital. Thus, we found that when intra-role conflict exists, it seemed that the job roles with less permeable boundaries (e.g., clinical) pushed out the job role with more permeable boundaries (i.e., research). However, given the salience of research to the hospital, university, and among faculty who internalize its importance, faculty are in a bind, as research tasks are left incomplete at the end of a long workday. As a result, faculty need to take their research work home, thereby interfering with time and obligations for home life, creating inter-role conflict. This pattern of findings suggests an importance contribution for role theory; namely that work-work (intra-role) conflict may be most likely to lead to work-life (inter-role) conflict when a highly salient work role has highly permeable boundaries. This possibility should be explored in future research.

Third, we found that job resources have a differential relation with work-life conflict, depending on the type of job resource. On the basis of these findings, we suggest an extension to the job demands-resources model: we propose that job resources can be classified as either *supportive*, in that they help people to manage demands and meet the requirements of their job, or as *stimulating*, because they help promote growth and development. There are parallels between stimulating job resources and motivating factors (e.g., achievement, recognition, challenging or stimulating work), which lead workers to feel high job satisfaction and achieve high performance in Herzberg et al.’s (1959) two-factor theory. Importantly, supportive job resources and stimulating job resources may have differential relations with outcomes.

On the one hand, consistent with the job demands-resources model (Demerouti et al., 2001), experiencing supportive job resources appear to directly decrease work-life conflict and to mitigate the negative effects of job demands on stress, work-life conflict, and exhaustion because they help to reduce or streamline medical faculty’s workload, thereby reducing work-life conflict. On the other hand, consistent with conservation of resources theory (Hobfoll, 1989, 2010), stimulating job resources appear to *increase* work-life conflict because stimulating job resources (e.g., challenging and varied work) can lead individuals to feel more empowered and engaged at work, which can lead them to reinvest their time and energy (e.g., by working longer), leading to greater work-life conflict, and eventual exhaustion (c.f., Nordhall et al., 2020). Thus, in contradiction with the job demands-resources model (Demerouti et al., 2001), we propose that work engagement can lead to ill-being. Future research should test these propositions and their generalizability. It may be the case that greater engagement leads to higher work-life conflict only among people who are highly involved and dedicated to their jobs, as are these medical faculty.

Fourth, our results suggest that, in the face of high stress and extreme work-life conflict, stimulating job resources may prevent the full experience of burnout. Although many respondents suffered from emotional exhaustion, no respondents expressed experiencing reduced self-efficacy or depersonalization—the other components of burnout. Despite heavy job demands, stimulating job resources (e.g., opportunities to make a difference in others’ lives), provide a sense of meaning and relatedness that may buffer against depersonalization. Similarly, stimulating job resources (e.g., varied work and opportunities to innovate) provide many faculty with a sense of competence and impact that seemingly buffers against any potential loss of self-efficacy. Thus, our results suggest that stimulating job resources may protect workers from depersonalization and reduced self-efficacy because they increase feelings of psychological empowerment (i.e., competence, autonomy, meaning, impact); however they may be insufficient to protect against exhaustion as well (c.f., Nordhall et al., 2020). However, it is also possible that the faculty experiencing full burnout did not volunteer to participate in our study.

### Strengths of the Current Research

A clear strength of the current study is that we investigated work-life conflict among medical faculty at a top research hospital whose multiple work roles, heavy job demands, and extensive resources, made them a highly pertinent sample. Importantly, we were able to achieve a decent sample size (*N* = 30), as well as saturation of our findings.

Using thematic analysis allowed us to derive a rich, nuanced understanding of the medical faculty’s work-life interface, well-being, and ill-being. It also allowed us to contextualize faculty responses within the culture of their institution and their profession. Finally, the use of thematic analysis allowed us to interpret the data in relation to relevant psychological theories and findings, garner unanticipated insights from the data, and emphasize similarities and differences across the interviews (Braun and Clarke, 2006).

We followed multiple verification procedures to ensure strong methodological rigor and trustworthiness of findings (Lincoln and Guba, 1985; Korstjens and Moser, 2018). We employed triangulation (e.g., use of multiple sources of data, analysts, theories), negative case analysis (e.g., searching for and incorporating data that does not support patterns that emerged through the data analysis), member checks (e.g., focus groups to review preliminary results with participants to gain their perspective), prolonged engagement (e.g., engaging in extended relationships with insiders and participants from developing the research questions and the interview guide to holding feedback sessions), and persistent observation (e.g., reading the interviews numerous times, analyzing them, and theorizing about them) in order to have greater confidence in our findings and interpretations (i.e., credibility; Lincoln and Guba, 1985; Korstjens and Moser, 2018).

We engaged in thick description of our data, that is, we describe the data with a high level of detail, so that others can determine if our findings are generalizable to other samples, contexts, and settings (i.e., transferability). We had participants and stakeholders evaluate and support the interpretations of the findings; thus, the findings of the research also have demonstrated confirmability. Detailed notes kept by R.L. and V.G. of the research steps from start to finish, as well as clear notes throughout the coding process also provide support so that the findings and interpretations are consistent and potentially replicable (i.e., dependability).

Finally, throughout the research process, discussions were held among authors, analysts, and stakeholders, who offered diverse feedback and perspectives through which to understand participants’ experiences. These varying perspectives helped R.L., V. G., and L.S.H. to confront biases and previously held assumptions and examine how they might affect our interpretations (i.e., reflexivity; Lincoln and Guba, 1985; Korstjens and Moser, 2018).

### Limitations and Future Research Directions

A limitation of the research is that our sample may not be representative of medical faculty at the hospital, other medical faculty, or physicians in general. The medical faculty who chose to be interviewed may have been more concerned with stress or work-life conflict than their colleagues. Further, those who were experiencing the most stress, work-life conflict, or burnout, would have been less likely to participate. In addition, given the job profiles of our sample (see Table 1), the data may better reflect the experiences of clinical-focused faculty. Further, some of our sample’s experiences may not generalize to medical faculty working at a less resource-rich, and potentially less-demanding hospitals. Nonetheless, many of the characteristics of the sample, such as high job demands, a great sense of purpose, and dedication are also found in other occupations. Thus, we propose that many our findings are likely to generalize to worker in occupations that people identify highly with, are highly involved in, and that are very demanding and intrinsically motivating, such as those in caring professions (e.g., nurses, first responders, teachers, social workers, veterinarians). Future research should test the transferability and generalizability of our research findings with other samples.

Another potential limitation of the current research is that the study was advertised to potential participants as a collaboration between the organization and external researchers. It is possible that participants, knowing that their results—at the aggregate level—would be shared with the hospital, performed as ideal employees, demonstrating high levels of commitment, engagement, and self-sacrifice. However, participants also expressed views that are not socially desirable in nature, such as criticism of hospital practices, scorn for those who complain about work-life conflict, and fears that lack of sleep may impact job performance. Thus, it appears that the interviewer was successful in conveying to participants that their individual data would be confidential and unidentifiable to hospital administration, thereby mitigating motives to respond in a socially desirable manner. It is also possible that participants may have been motivated to amplify the nature of work-life conflict in an attempted plea to hospital administration for change. However, many participants also expressed views that the *status quo* is fine, such as those who reported low levels of work-life conflict, rejected the notion of work-life conflict, justified high work demands, and all who said how much they loved their work. Thus, it appears that not all participants treated the interviews as a forum to vent frustrations and prompt change.

Further, the interviews were conducted with faculty in their offices at the hospital, for participants’ convenience. It is possible that the location of the interviews, heightened the salience of their work role and the hospital and its culture, which may have hindered faculty’s ability to consider and articulate facilitative processes between their work and home. Observational research may be a useful methodology for future research because participants could be observed in both work and home settings.

There are a few disadvantages of using thematic analysis. First, though the flexibility of thematic analysis is often touted as a strength (Braun and Clarke, 2006), it can also lead to greater inconsistency in analyses, such that different codes and themes would be elicited with different researchers. Second, there is a lack of clear agreement from researchers regarding steps or guides on how to engage in thematic analysis, compared with other qualitative methods (e.g., ethnography, grounded theory; Nowell et al., 2017). However, we followed existing thematic analysis guides that have been gaining popularity and support (i.e., Braun and Clarke, 2006) and we followed recommended qualitative practices to ensure that our methodology was as rigorous as possible (e.g., Lincoln and Guba, 1985).

### Practical Implications

Given the role of job demands in leading to work-life conflict and the potential for supportive job resources to lessen it, hospital leadership should do what they can to reduce unnecessary job demands and to increase supportive job resources. On the basis of our findings, we recommend that organizations (hospitals and universities) reduce overly burdensome demands, such as poor IT (e.g., outdated computers and software) and performance evaluations that are too frequent and overly focused on one work role. More specifically, we recommend that excellent performance for *all aspects* of medical faculty’s jobs (e.g., clinical work) should be accurately assessed and rewarded through fair evaluation systems administered not more than once every 2 years, and through informal support and recognition. In addition, hospitals should aim to build supportive job resources, such as well-trained staff, excellent residents, and ease of schedule flexibility. These changes should help faculty reduce some of the burdens of their workload so they may live more balanced lives. Organizations should also seek to support medical faculty to shape their jobs to be highly stimulating so that feelings of meaning and impact can stave off experiences of burnout. For instance, as much as possible, faculty should be given the physical (e.g., space, equipment), financial, and time resources they need in order to be able to develop new expertise and conduct research in novel ways.

Almost all of the medical faculty we interviewed believe that to be maximally productive and to perform at the level of excellence expected of them, they need to sacrifice their own self-care, well-being, family time, and life outside of work. As such, hospitals, and organizations more broadly, should examine whether such trade-offs are in line with their organizational mission and values, and if they are valid assumptions for their employees to hold. To help realize *healthcare*, hospitals (and other organizations), should promote a culture in which a dedication to excellence is not put at odds with a commitment to wellness for staff. Rather, there needs to be formal recognition by administrators and physicians themselves that, for example, performance depends upon adequate sleep (Krueger, 2007), creativity at work is related to exercise (Steinberg et al., 1997), and exceptional care of patients is contingent upon the mental well-being of physicians (Shanafelt et al., 2002). There should be recognition that, for many medical faculty, there is great stimulation and reward found in their work, and as such, it is difficult for them to pull themselves away from it. Thus, for some, the motivation to take breaks may come from a desire for work-life balance, but for others, the motivation may come from an awareness that breaks are needs for restoration and higher work performance.

As well, hospitals, and in particular, supervisors, need to develop more explicitly supportive work-life climates and encourage work-life facilitation. These messages could be reinforced through organizations’ learning and development programs, which could expand their focus to consider holistic development inside and outside of work. In addition, given that the vast majority of our participants seem to have wholeheartedly adopted a belief that work should always come before themselves and their non-work lives, it is likely necessary to target these issues as early as possible in their medical training (i.e., at medical school). In addition, organizations can work to combat against physician burnout by: explicitly including burnout as an indicator of well-being in engagement surveys, assessing burnout at the team- or department-level to understand underlying contributors, and encouraging input from staff and patients to gain deeper and holistic insights regarding physician wellness (Montgomery et al., 2019).

Among our participants, men and women were equally likely to describe high levels of work-life conflict, which is consistent with previous meta-analytic research on workers in general (Shockley et al., 2017). However, also consistent with previous research (Borelli et al., 2017), we found that women faculty reported experiencing guilt as a result of their prioritization of work over family or home more than men faculty. In addition, only women discussed changing their job profile to be less research intensive and reducing their work hours due to work-life conflict. Given the differential home and family demands placed on men and women, which may be further exacerbated due to COVID-19, as women are expected to experience a “greater burden of this pandemic” (Malisch et al., 2020), we beseech hospitals (and other organizations), to create performance evaluations systems that recognize these differences. Specifically, candidates should be given the opportunity to identify the issues in their personal and family life that may be impacting their productivity and performance at work. Further, the committees that oversee these evaluations must be trained to view this information as context to help inform accurate and fair assessments of performance, rather than as information to hold against the candidate. In doing so, performance evaluations take into consideration all relevant aspects of a candidate’s life that may affect their work and help to ensure that people are being fairly judged. Further, extra care should be taken that female faculty are not encouraged more than male faculty to reduce their research role or go to part-time status to manage their work-life conflict, especially because such decisions will contribute to the gender gap in faculty pay (Johnson and Taylor, 2019), delay or impede women’s promotion to full professor (Winslow and Davis, 2016), and reinforce gender stereotypes about women’s lack of dedication to work (Ellemers et al., 2004).

Additionally, our research and findings have important implications for organizations that employ engagement surveys or other self-report measures, as well as for researchers who use these methods. Two of our findings call into question the ability of self-report survey methods to capture the work-life interface as researchers intend. First, our findings demonstrate that some faculty believed that their positive experiences at work must enrich their home lives (and vice versa), yet they had difficulty describing such experiences. If these faculty’s inability to articulate work-life facilitation reflects a lack of lived experience, they would presumably score high on a self-report measure of work-life facilitation (due to wishful platitudes), and yet, these reports likely would not predict the positive outcomes that should be associated with high work-life facilitation (e.g., job satisfaction; McNall et al., 2010). Thus, the validity of self-report survey measures of work-life facilitation may be questionable for some respondents.

Second, we also discerned a number of cognitive reappraisal strategies used by some of the participants to reduce some of the negative consequences of work-life conflict: normalization of work-life conflict, legitimization of high work demands as normal or expected, and denial that work and non-work domains should be separate. Using traditional survey methods, these faculty could potentially score low on work-life conflict, yet their own stories may belie the veracity of such a report. These faculty’s uses of such coping mechanisms demonstrate the importance of investigating these issues with methods other than self-report surveys, as they would be unable to detect such processes. We recommend that practitioners and researchers employ a mixed-methods approach to study these phenomena, using self-report surveys (e.g., Sancassiani et al., 2015; Galletta et al., 2016; Carta et al., 2017), as well as qualitative and 360-degree feedback to gather a more nuanced and in-depth sense of these experiences for workers.

## Conclusion

The findings of the current research shed some light on how and why medical faculty may experience high levels of job satisfaction and work engagement (i.e., well-being) but also high levels of stress and emotional exhaustion (i.e., ill-being). Many medical faculty interviewed experience unmanageable levels of job demands that appear to coalesce and work in conjunction to force them to work long hospital hours and bring work home, and their high levels of work-life conflict had damaging effects on stress, exhaustion, self-care, and relationships with loved ones. However, they also enjoy very stimulating jobs that provide a sense of meaning and impact that motivates them to be highly engaged with their work. In order for current and future physicians to function and integrate their non-work and work lives, hospitals and the medical field more broadly may need to temper the culture of excellence and research productivity with one that also truly values physicians’ well-being, families, and lives outside of work.

## Data Availability Statement

The datasets presented in this article are not readily available because the qualitative interview data include statements that are potentially identifying. Requests to access the datasets should be directed to Leanne Son Hing who can provide aspects of the data with identifying information removed.

## Ethics Statement

The study involving human participants was reviewed and approved by the University of Guelph Research Ethics Board and the Hospital’s Research Ethics Board. The participants provided their written informed consent to participate in this study.

## Author Contributions

RL, LSH, and VG: conception and design of the study. RL, LSH, VG, and SW: interview guide creation. RL, LSH, VG, SW, and DD: acquisition of data. RL, LSH, VG, DL, and PH: analysis and interpretation of data. All authors contributed to drafting the article and revising it. In addition, all authors have given final approval and have agreed to be accountable for all aspects of the work in ensuring that questions related to the accuracy or integrity of any part of the work are appropriately investigated and resolved.

## Conflict of Interest

The authors declare that the research was conducted in the absence of any commercial or financial relationships that could be construed as a potential conflict of interest.

## Appendix

### Interview Guide

(1) Can you describe the work that you do? What role do you play in the hospital?
  • Probe for rank, role (i.e., academic clinician, clinician-scientist, clinician-investigator), full/part-time, and tenure
(2) Please describe some of the faculty members that are admired the most around here. Why do people feel this way about them? There is no need for you to use names.
(3) How do you feel about your job and the work that you do? What do you like most about it? What do you like least? Can you give an example?
(4) What were you hoping your work would look like when you started here, and has it lived up to that?
(5) What about your work is particularly motivating for you? What gets you excited? Can you give me an example? How do you think this affects your well-being in general?
(6) How stressful do you find your work to be? What is particularly stressful for you? Can you give me an example? How do you think this affects your well-being in general?
  • Probe for strain, physical and psychological effects
(7) Do you feel that you are successful at your job? How do you think this affects your wellbeing?
(8) Can you tell me about how the evaluation and promotion process works here? When was your last conversation with the program about career development and compensation? Do you believe the process is fair?
(9) What about promotions within the university? Are both positions (hospital and university) valued and respected?
(10) What are some of the challenging demands at work that make it more difficult to achieve your work goals? These could be personal, or job-task related. It could include things like workload, undesirable or difficult tasks, or things of a more social or emotional nature.
(11) Are there other demands that are really challenging for other people?
  • Follow-up: Who are you talking about?
(12) Do some people face more demands than others? To what extent are they unequally distributed? Can you give an example? Do you feel this is appropriate? Why or why not?
(13) What determines the demands each person faces? Do you think this is fair? Do you think that any group memberships come into play in terms of who gets what? (e.g., gender or ethnicity) Why or why not?
  • Follow-up: Who are you talking about?
(14) What personal needs or goals does work help you to fulfill? What kind of resources can you access at work that makes it easier for you to achieve your work goals? These could be personal, or job-task related. Think of things the organization or leaders can give you to make it easier for you to do your work, like material resources or social resources.
(15) Are there other resources that are really important for other people?
  • Follow-up: Who are you talking about?
(16) Do some people have more resources than others? To what extent are they unequally distributed? Can you give an example? Do you feel this is appropriate? Why or why not?
(17) What determines how much essential resources people have? Do you think this is fair? Do you think that any group memberships come into play in terms of who gets what? (e.g., gender or ethnicity) Why or why not?
  • Follow-up: Who are you talking about?
(18) Do you have any young children or other relatives that you are caring for?
(19) Are there any other major commitments in your personal life that create a drain on your time or resources?
(20) Does your work interfere with your non-work life? In what way? Can you give me an example? How many hours do you work at the hospital, and how much do you have to work at home? What effect does this have on you and your family?
(21) Does your non-work life interfere with your work? In what way? Can you give me an example? What effect does this have on you and your family?
(22) Can you tell me about some specific strategies that you use to cope with the stress caused by your job or by work-life interference? What resources do you draw on to help with work-life interference?
(23) What resources outside of work do you draw on to help manage demands in your personal life?
(24) Have you used any of the hospital’s wellness-related programs or services (e.g., book club, gym)? If so, how effective are they?
(25) Do you think these issues are inevitable, or could something be done to improve it? What do you think could be done?
(26) What do you find more stressful, demands at work or demands at home? Why? How do you find yourself dealing with demands at work versus demands at home? Are you more successful with one over the other?
(27) Have your work and non-work lives had any positive effect on each other? Can you provide an example?
(28) When responding to the questions today, who were you thinking about: people in the hospital in general, just the faculty, just your department, just your division? Or some blend?
(29) How do people in X (*depending on answer to previous question)* get along? Can you give examples?
(30) Is there anything else that you would like to share about the areas we’ve discussed today?
